# A quantum engine in the BEC–BCS crossover

**DOI:** 10.1038/s41586-023-06469-8

**Published:** 2023-09-27

**Authors:** Jennifer Koch, Keerthy Menon, Eloisa Cuestas, Sian Barbosa, Eric Lutz, Thomás Fogarty, Thomas Busch, Artur Widera

**Affiliations:** 1 Department of Physics and Research Center OPTIMAS, RPTU Kaiserslautern-Landau, Kaiserslautern, Germany; 2 OIST Graduate University, Onna, Japan; 3 Enrique Gaviola Institute of Physics, National Scientific and Technical Research Council of Argentina and National University of Córdoba, Córdoba, Argentina; 4 Institute for Theoretical Physics I, University of Stuttgart, Stuttgart, Germany

**Keywords:** Quantum physics, Thermodynamics, Ultracold gases

## Abstract

Heat engines convert thermal energy into mechanical work both in the classical and quantum regimes^1^. However, quantum theory offers genuine non-classical forms of energy, different from heat, which so far have not been exploited in cyclic engines. Here we experimentally realize a quantum many-body engine fuelled by the energy difference between fermionic and bosonic ensembles of ultracold particles that follows from the Pauli exclusion principle^2^. We employ a harmonically trapped superfluid gas of ^6^Li atoms close to a magnetic Feshbach resonance^3^ that allows us to effectively change the quantum statistics from Bose–Einstein to Fermi–Dirac, by tuning the gas between a Bose–Einstein condensate of bosonic molecules and a unitary Fermi gas (and back) through a magnetic field^4–10^. The quantum nature of such a Pauli engine is revealed by contrasting it with an engine in the classical thermal regime and with a purely interaction-driven device. We obtain a work output of several 10^6^ vibrational quanta per cycle with an efficiency of up to 25%. Our findings establish quantum statistics as a useful thermodynamic resource for work production.

## Abstract (web summary)

This study reports the creation of a model thermodynamic engine that is fuelled by the energy difference resulting from changing the statistics of a quantum gas from bosonic to fermionic.

## Main

Work and heat are two fundamental forms of energy transfer in thermodynamics. Work corresponds to energy change at constant entropy, as in the case of the variation of the position of a piston, whereas heat exchange necessarily causes entropy increase^1^. From a microscopic point of view, work corresponds to a displacement of energy levels and heat to a modification of their level probability distribution by contact with a thermal bath, for constantly driven quantum systems^11^. In other cases, this distinction may not always hold^12–14^. Quantum heat engines realized so far convert thermal energy into mechanical work by cyclically operating between effective thermal reservoirs at different temperatures^15–20^ like their classical counterparts, where heating and cooling strokes redistribute the quantum state populations^21^. However, owing to the existence of distinct particle (Fermi or Bose) statistics, the level occupation probabilities of quantum many-body systems may strongly differ at the same temperature^1^. Changing the quantum statistics would thus lead to a new, purely quantum, form of energy transfer.

At ultralow temperatures, in the quantum-degenerate regime, an ensemble of indistinguishable bosonic particles will all accumulate in the ground state, whereas fermionic systems will occupy quantum states with increasing energy due to the Pauli exclusion principle^2^. These different quantum statistical behaviours are intimately linked to the symmetry of the many-body wavefunctions and originate from the spin-values of the particles. They also result in a profound energy difference between the two particle classes and the emergence of a degeneracy pressure in the Fermi systems. The exclusion principle plays an essential role for the stability of matter^22^ and the physics of stars^23^. A change in quantum statistics may be experimentally achieved in interacting atomic Fermi gases using Feshbach resonances^3^. In such systems, the crossover between a Bardeen–Cooper–Schrieffer (BCS) state of pairs of fermions to a molecular Bose–Einstein condensate (BEC) state of diatomic bosonic molecules can be realized by tuning an external magnetic field^4–10^.

**Fig. 1:**
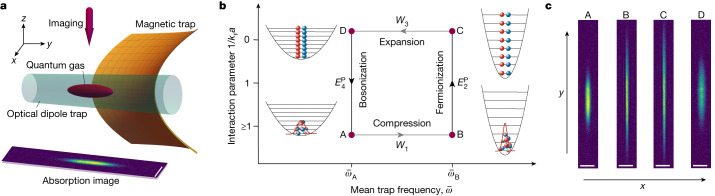
Principles of the quantum Pauli engine. **a**, Schematic of the experimental set-up. The atom cloud (purple ellipsoid) is trapped in the combined fields of a magnetic saddle potential (orange surface) and an optical dipole trap potential (blue cylinder) operating at a wavelength of 1,070 nm. The absorption pictures are taken with an imaging beam (purple arrow) in the −*z* direction. The scale bar on the absorption picture corresponds to 50 μm. **b**, Cycle of the Pauli engine. Starting with a molecular BEC that macroscopically populates the ground state of the trap at well-defined temperature *T* (point A), the first step, A → B, performs work *W*_1_ on the system by compressing the cloud through an increase of the radial trap frequency ${\bar{\omega }}_{{\rm{B}}} > {\bar{\omega }}_{{\rm{A}}}$. This is achieved by enhancing the power of the trapping laser. The second stroke, B → C, increases the magnetic field strength from *B*_A_ = 763.6 G (76.36 mT) to the resonant field *B*_C_ = 832.2 G, while keeping the trap frequency constant. This leads to a change in the quantum statistics of the system as the working medium now forms a Fermi sea with an associated addition of Pauli energy ${E}_{2}^{{\rm{P}}}$, which substitutes the heat stroke. Step C → D expands the trap back to the frequency ${\bar{\omega }}_{{\rm{A}}}$ and corresponds to the second work stroke *W*_3_. Finally, the system is brought back to the initial state with bosonic quantum statistics during step D → A by reducing *B*_C_ to *B*_A_, which corresponds to a change in the Pauli energy ${E}_{4}^{{\rm{P}}}$. The population distributions in the harmonic trap of the atoms with spin up (blue) and spin down (red) are indicated at each corner. **c**, Examples of absorption pictures at each point of the engine cycle, where the particular change in size from B → C is due to the Pauli stroke indicating that the Pauli energy increases the size of the cloud in the external potential. Scale bars, 50 μm.

Here, we report the experimental realization of a new many-body quantum engine (‘Pauli engine’) where the temperature variation, usually induced through coupling to a hot or cold thermal bath, is replaced by a change of quantum statistics of the system, from Bose–Einstein to Fermi–Dirac (and back). This engine cyclically converts energy stemming from the Pauli exclusion principle (‘Pauli energy’) into work. Its mechanism is of purely quantum origin, since the difference between fermions and bosons disappears in the classical high-temperature limit. We specifically employ an ultracold two-component Fermi gas of ^6^Li atoms confined in a combined opto-magnetic trap^24^ (Fig. 1), prepared close to a magnetic Feshbach resonance. Inspired by the quantum Otto motor^25^, we implement a cycle by adiabatically varying the trap frequency by means of the power of the laser forming the trapping potential, and hence perform work. We further adiabatically change the magnetic field through the crossover at constant trap frequency, which thus changes the quantum statistics and the associated occupation probabilities. This step leads to the exchange of Pauli energy instead of heat. We emphasize that all strokes can, in principle, be described by Hamiltonian dynamics, which preserves the entropy of the system throughout the cycle, in contrast with conventional quantum heat engines^15–21^. We measure atom numbers and cloud radii from in situ absorption images^26^, from which we determine the energy of the gas after each stroke. We employ the latter quantities to evaluate both efficiency and work output of the Pauli engine, analyse its thermodynamic performance when parameters are varied and compare it with theoretical calculations.

It is instructive to begin with a discussion of the simple case of a one-dimensional (1D) harmonically trapped non-interacting ideal gas at zero temperature to gain physical insight into the energetic possibilities of the Pauli principle^2^. A fully bosonic system only populates the ground state with energy *E*^B^ = *Nħω*/2, where *N* is the number of particles, *ħ* is the reduced Planck constant and *ω* is the trap frequency^1^. By contrast, the fermionic counterpart populates all the energy levels up to the Fermi energy *E*_F_ = *ħω*(2*N* − 1)/2 and the total energy of the system is accordingly *E*^F^ = *ħωN*^2^/2 (ref. ^1^). The resulting energy difference due to the change of quantum statistics, the Pauli energy, is therefore *E*^P^ = *E*^F^ − *E*^B^ = *ħωN*(*N* − 1)/2. This enormous difference of total energy at zero temperature originates from the underlying quantum statistics, dictating a population probability distribution across the available quantum energy levels *E*_*n*_ according to ${f}_{n}=1/[\exp [({E}_{n}-\mu )/({k}_{{\rm{B}}}T)]\pm 1]$, where the + (−) sign in the denominator is for fermions (bosons) with half-integer (integer) spin^27^; here, *μ* is the chemical potential and *k*_B_ is the Boltzmann constant. For increasing temperature, both distributions reduce to the classical Boltzmann factor. Microscopically, the Pauli energy is equal to *E*^P^ = ∑_*n*_Δ*f*_*n*_*E*_*n*_, an expression reminiscent of that of heat for systems coupled to a bath^11^. Importantly, the quadratic dependence of the energy difference on the particle number between BEC and Fermi sea in the 1D case implies that the Pauli energy can be substantial for large *N*, far exceeding typical energy scales in comparable quantum thermal machines. The influence of the quantum statistics on work production has been theoretically discussed in refs. ^28–30^ and a quantum heat engine operating across a BEC phase transition has been considered in ref. ^31^.

We prepare an interacting, three-dimensional (3D) quantum-degenerate two-component Fermi gas of up to *N* = 6 × 10^5^^6^ Li atoms (Fig. 1a) (for details see ref. ^32^), with equal population *N*^*i*^ = *N*/2 of two lowest-lying Zeeman states *i*. A broad Feshbach resonance centred at ${B}_{{\rm{res}}}=832.2\,{\rm{G}}(=83.22\,{\rm{mT}})$ (ref. ^33^) allows us to change the nature of the many-body state: a molecular BEC of *N*/2 molecules is formed at magnetic field strengths below the resonance, whereas on resonance a strongly interacting Fermi sea of *N* ^6^Li atoms emerges.

For the bosonic regime, we operate at *B*_A_ = 763.6 G = *B*_B_, where the molecular BEC has an interaction parameter of 1/*k*_F_*a* ≈ 2.3; *k*_F_ denotes the magnitude of the Fermi wavevector and $a={a}_{{\rm{b}}{\rm{g}}}[1-\varDelta /(B-{B}_{{\rm{r}}{\rm{e}}{\rm{s}}})]$ is the *s*-wave scattering length, with the background scattering length *a*_bg_ and the resonance width *Δ* (ref. ^3^). The temperature of the gas is about *T* ≈ 120 nK, corresponding to *T*/*T*_F_ ≈ 0.3 with Fermi temperature ${T}_{{\rm{F}}}=\hbar \bar{\omega }{(3N)}^{1/3}/{k}_{{\rm{B}}}$, where $\bar{\omega }$ is the geometric mean trap frequency, which can be experimentally controlled. The unitary regime (which saturates the unitarity bound of the scattering matrix) appears on resonance, *B*_C_ = 832.2 G = *B*_D_. In this limit, the gas is dilute, but strongly interacting, and exhibits universal behaviour, which is independent of the microscopic details owing to the divergence of the scattering length, 1/*k*_F_*a* = 0 (refs. ^10,34^). Pauli blocking of occupied single-particle states here leads to Fermi–Dirac-type statistics^35–38^. However, it is also possible to change the magnetic field and, therefore, the interactions, without changing the statistics by staying away from the resonance. When the molecular BEC is adiabatically ramped to unitarity, the reduced temperature drops to values well below *T*/*T*_F_ < 0.2 (ref. ^39^).

The experimental implementation of the Pauli cycle ABCD starts with a molecular BEC (Fig. 1b). It consists of four strokes: compression, fermionization, expansion and bosonization. We first analyse the Pauli stroke B → C during which the change of quantum statistics takes place. Contrary to an ideal gas, atoms in the experiment are not at zero temperature and further interact with a strength that depends on the magnetic field. All of these have an effect on the cloud size.

**Fig. 2:**
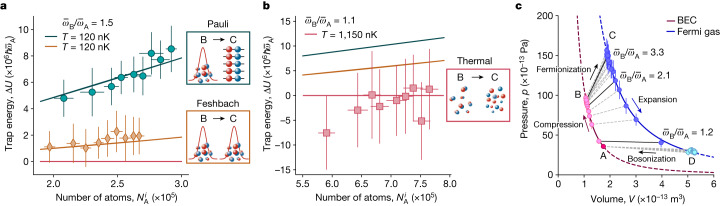
Contribution of the Pauli energy. **a**,**b**, Trap energy variation Δ*U* as a function of the number of atoms in a single spin state ${N}_{{\rm{A}}}^{i}$ for the magnetic field change between cycle points B → C for a gas performing a Pauli stroke (bringing the gas from a molecular BEC to a unitary gas, cyan), a Feshbach stroke (always remaining in the molecular BEC regime, orange) and a thermal stroke for the same magnetic fields as in the Pauli stroke (red). Symbols represent mean values of 20 repetitions; solid lines are predictions of our model. Owing to the much increased temperature of the gas in the thermal case, trap depth and compression ratio have been chosen differently in the experimental realization to be for the Pauli and Feshbach strokes: *T* ≈ 120 nK, *T*/*T*_F_ ≈ 0.3 (measured in the molecular BEC regime) and ${\bar{\omega }}_{{\rm{B}}}/{\bar{\omega }}_{{\rm{A}}}=1.5$ (**a**); and for the thermal stroke: *T* ≈ 1,150 nK, *T*/*T*_F_ ≈ 0.7 (measured in the molecular BEC regime) and ${\bar{\omega }}_{{\rm{B}}}/{\bar{\omega }}_{{\rm{A}}}=1.1$ (the higher temperature also means that fewer atoms are lost by evaporation) (**b**). The insets show microscopic sketches of the quantum state of the gas, transitioning between the molecular BEC and Fermi sea, remaining in the molecular BEC, or transitioning between a gas of free molecules to a gas of free atoms, respectively. The error bars for all data points denote 1*σ* statistical fluctuations of 20 repetitions. **c**, Pressure–volume (*p–V*) diagram of the Pauli engine for different compression ratios (black solid and dashed lines). The blue (red) line indicates the equation of state of the Fermi gas (molecular BEC). Varying the compression ratio moves the state of the gas along each line, effectively changing the engine points B and C, whereas the Pauli strokes (bosonization and fermionization) induce transitions between the red and blue lines.

For a harmonically trapped interacting Fermi gas, total energy *E* and trap energy *U* are related by means of the generalized virial theorem $E=2U-\hbar {\mathcal{I}}/(8\pi am)$, with the contact ${\mathcal{I}}$ and the mass *m* of a ^6^Li atom^40–42^. For a resonantly interacting gas, the contact vanishes and *E* = 2*U*. Using the radial symmetry of the trap, with radial frequencies *ω*_*x*_ ≈ *ω*_*z*_, the trap energy is given by $2U=N(2m{\omega }_{x}^{2}\langle {x}^{2}\rangle +m{\omega }_{y}^{2}\langle {y}^{2}\rangle )$, where ⟨*x*^2^⟩, ⟨*y*^2^⟩ are the mean-square sizes of the cloud in the *x*, *y* directions, determined by means of in situ absorption images. In the molecular BEC regime, the total energy additionally comprises the molecular binding energy and the residual particle–particle interaction (Methods). The binding energy does not contribute to work production and is thus omitted. To isolate the contributions to the cloud size coming from the change of statistics (Pauli energy) and from the change of the residual particle–particle repulsion with the magnetic field, we compare, in Fig. 2a, the energy difference Δ*U* for a Pauli stroke (where interaction strength and quantum statistics are both changed by moving to the fermionic regime) with a so-called Feshbach stroke, where we change the interaction strength but effectively remain in the bosonic regime^43,44^. We further contrast the quantum Pauli stroke with a similar stroke in the thermal regime, with the same magnetic field ramp, at a much higher temperature of *T*/*T*_F_ ≈ 0.7 in Fig. 2b to stress the quantum nature of the effect. We observe that the change of quantum statistics during the Pauli stroke yields a much larger energy difference Δ*U* and a much faster increase with the particle number compared with the two other strokes. Hence, we may conclude that the variation Δ*U* is mostly due to the quantum Pauli energy *E*^P^. An increase of turn-over energy in the quantum degenerate compared with the thermal regime was also recently observed in ref. ^45^. We also note good agreement with the theoretical curves (solid lines) obtained using analytical formulas in the Thomas–Fermi regime for the molecular BEC^35,46^ and the known energy expressions for a strongly interacting Fermi gas at unitarity^34,35^ (Methods).

The exclusion principle not only affects the energy but also the pressure of a quantum system: the pressure of a degenerate Fermi gas is indeed non-zero at zero temperature in contrast to that of a Bose gas^1^. From that perspective, the Pauli engine reduces the pressure of the Fermi gas at constant entropy by converting it into a molecular BEC and exploits the fact that work done on the gas during compression is smaller than during expansion because of its different dependence on the number of atoms. The pressure of the working medium may be defined as *p* = −(∂*E*/∂*V*) (ref. ^1^), with the cloud’s volume *V* determined from the measured radii (Methods). The corresponding work along one branch is then given by the familiar expression −∫ *p*d*V*. The experimentally reconstructed *p–V* diagram is shown in Fig. 2c. As our engine does not require coupling to an external bath, it differs from any quantum machine experimentally realized so far^21^.

**Fig. 3:**
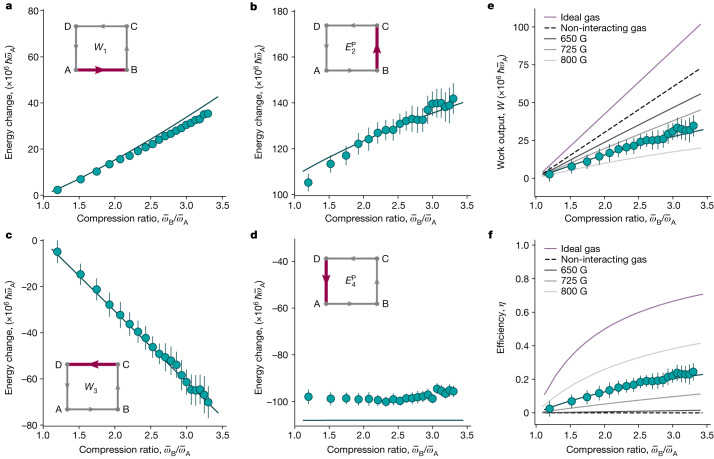
Performance of the Pauli engine. **a**–**d**, Work contributions, *W*_1_ (**a**) and *W*_3 _(**c**), and Pauli energies, ${E}_{2}^{{\rm{P}}}$ (**b**) and ${E}_{4}^{{\rm{P}}}$ (**d**), as a function of the compression ratio ${\bar{\omega }}_{{\rm{B}}}/{\bar{\omega }}_{{\rm{A}}}$ for fixed ${\bar{\omega }}_{{\rm{A}}}$ and fixed number of atoms ${N}_{{\rm{A}}}^{i}\approx 2.5\times 1{0}^{5}$ at point A. The experimental data points (cyan dots) are the mean value of 20 repetitions and the error bars indicate their 1*σ* statistical uncertainty. The numerical calculations are indicated by the (cyan) solid lines. The insets show the corresponding stroke. **e**,**f**, Work output *W* (**e**) and efficiency *η* (**f**). The experimental points and the numerical simulations are in cyan. For comparison, the *W* and *η* values for a non-interacting gas are depicted as black dashed lines. They have been obtained by setting the *s*-wave scattering length *a* to zero for points A and B (this limit corresponds to a magnetic field *B*_A_ far below the resonance, leading to point-like composite bosons with infinite binding energy) and using the formulas for the energy at unitarity for points C and D. Also, *W* and *η* for an ideal gas are shown as purple solid lines and mark the upper bounds of the machine. They have been obtained by using the energy of ideal gases in the degenerate regime (Bose gas for points A and B and Fermi gas for points C and D). The grey solid lines are the theoretical values obtained for different magnetic fields: *B*_A_ = 800 G, 725 G and 650 G from lighter to darker (dimer–dimer interaction strength *g*/*g*_0_ = 2.53, 0.51 and 0.16, respectively, with *g*_0_ being the interaction strength for *B*_A_ = 763.3 G).

To experimentally evaluate the performance of the Pauli engine, we extract the energy change for every cycle stroke, by determining the clouds’ radii in the trap from absorption images (Methods and Fig. 3). The work output *W* and the efficiency *η* may then be computed in analogy with standard quantum engines^21^ as

1 $$W=-\left(\,{W}_{1}+{W}_{3}\right)\,\text{and}\;\eta =W\,/{E}_{2}^{{\rm{P}}},$$

where the Pauli energy ${E}_{2}^{{\rm{P}}}$ replaces the heat input *Q*_2_ in the efficiency. This is the main difference from a thermally driven engine. As with all quantum engines built so far^15–21^, the produced work is calculated, since the coupling to a work load is experimentally challenging to realize.

The energy changes for individual strokes (Fig. 3a–d) allow one to obtain an intuitive understanding of the performance. The two work strokes (Fig. 3a) and (Fig. 3c) simply follow the variation of the trap frequency. The important Pauli-stroke energy change (Fig. 3b) directly reflects the growing Fermi energy in steeper potentials for constant particle numbers, pointing towards increased work output for increased compression ratio. The complementary stroke (Fig. 3d) is independent of this change because the trap frequency ${\bar{\omega }}_{{\rm{A}}}$ is fixed in the experiment. The difference of about 10% between theory and data seen in stroke (Fig. 3d) is mainly due to the evaporation of hot molecules in the relatively shallow trap.

We observe that the work output *W* and the efficiency *η* both increase with the compression ratio (Fig. 3e–f), with a total work output of up to $30\times 1{0}^{6}\,\hbar {\bar{\omega }}_{{\rm{A}}}$ and an efficiency above 10% for compression ratios larger than 1.5. The largest efficiency achieved in the experiment is 25%, whereas, for a very large compression ratio of about 10 (not accessible in our experiment), the theoretical efficiency for the same experimental parameters can be higher than 50%. With a cycle time of *t*_cyc_ = 1.6 s, the power *P* = *W*/*t*_cyc_ is finite with values of up to 10^−24^ J s^−1^. The power could be increased by driving the Pauli engine diabatically, being then fundamentally limited by quantum speed limits^47^. However, non-adiabatic transitions are expected to suppress the efficiency^15–21^. Moreover, increased atom losses would further reduce the overall performance.

**Fig. 4:**
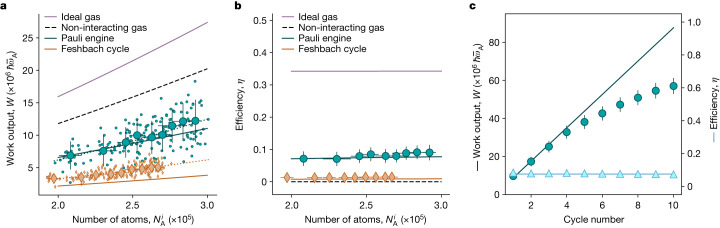
Comparison of the performance of quantum many-body devices. **a**,**b**, Work output (**a**) and efficiency (**b**) as a function of the number of atoms ${N}_{{\rm{A}}}^{i}$ in one spin state for a compression ratio ${\bar{\omega }}_{{\rm{B}}}/{\bar{\omega }}_{{\rm{A}}}=1.5$ for the Pauli engine (cyan dots) and the Feshbach cycle (orange diamonds). Small symbols denote individual realizations; large symbols indicate mean values of 20 repetitions with error bars indicating 1*σ* statistical fluctuations. For comparison, calculations for a non-interacting gas (black dashed line) and an ideal gas (purple solid line) are shown. Dotted lines are fits to the respective work output *W* ∝ *N*^*α*^. The fitted exponents *α* are 1.73(18) and 1.58(13) for the Pauli engine and Feshbach cycle, respectively, well reproduced by our theoretical model (solid cyan and orange lines). Importantly, the efficiency of the Feshbach cycle is essentially zero. **c**, Accumulated work output (cyan dots) and efficiency (blue triangles) of the Pauli engine over several cycles for a compression ratio ${\bar{\omega }}_{{\rm{B}}}/{\bar{\omega }}_{{\rm{A}}}=1.5$ and an initial number of atoms in one spin state of about *N*^*i*^ = 2.5 × 10^5^.

In addition, we note that *W* increases with the number of atoms as well as with the number of consecutive cycles (Fig. 4a–c), whereas *η* remains constant (at about 7% for the chosen parameters to limit atom losses that lower the work output). An additional increase of *W* with atom number can be expected in reduced dimensions, where the degeneracies in the trap are less. The comparison between the quantum-statistics-driven Pauli cycle and the interaction-driven Feshbach cycle in Fig. 4a,b shows that the Pauli engine outperforms the Feshbach engine, whose efficiency is essentially zero. Finally, the produced work scales as *W* ∝ *N*^*α*^ with *α* = 1.73(18), which is close to the theoretical prediction of 1.4 (Fig. 4a), which is different from the value one finds for a non-interacting classical gas^48^.

Thermodynamics is primarily the study of energy and its transformations. While most investigations of quantum thermodynamics have focused so far on conventional energy forms, such as work and heat^21^, we have shown that, by changing the quantum statistics of a system, a new form of energy transfer, which cyclically converts Pauli energy into work, can be realized. This effect, based on the Pauli exclusion principle, is intrinsically quantum. The Pauli energy associated with such a modification of quantum statistics may be very large: in solids, the energy of electrons in the conduction band corresponds to thousands of kelvin, much above the usually accessible thermal energies^49^. At the same time, since the operation of the Pauli engine follows, in principle, Hamiltonian dynamics owing to the absence of heat baths, it is not subjected to common friction mechanisms of quantum motors^21^. This could lead to energy-efficient quantum machines, including both engines and refrigerators. We further emphasize that the change of quantum statistics is a widespread phenomenon in many-body quantum systems (many bosons are actually composed of fermions). Cooper pairing plays, for instance, an essential role in superconductivity and superfluidity in condensed-matter systems^50^, which, unlike Feshbach resonances in cold-atom systems, do not require a tunable magnetic field. Exploring the exotic properties and the potential applications of this unconventional form of quantum energy transfer appears to be a fascinating prospect.

## Methods

### Set-up and sequence

We prepare a degenerate two-component fermionic ^6^Li gas in the two lowest-lying Zeeman substates of the electronic ground state ^2^*S*_1/2_ confined in an elongated quasi-harmonic trap comprising a magnetic and an optical dipole trap (ODT) operating at a wavelength of 1,070 nm; for details of the experimental set-up, see ref. ^32^. We cool the gas using evaporative cooling until reaching a temperature of 120 nK in a trap with geometric mean trap frequency $\bar{\omega }={({\omega }_{x}{\omega }_{y}{\omega }_{z})}^{1/3}$, where typical values used for the engine performance are given in Extended Data Table 1. Evaporation takes place on the BEC side of the crossover at a magnetic field of 763.6 G. After evaporation, we hold the cloud for 150 ms to let it equilibrate. By varying the loading time of the magneto-optical trap before evaporation, we adjust the number of atoms per spin state *N*^*i*^ of the cloud from 1.75 × 10^5^ up to 3.0 × 10^5^.

**Extended Data Fig. 1:**
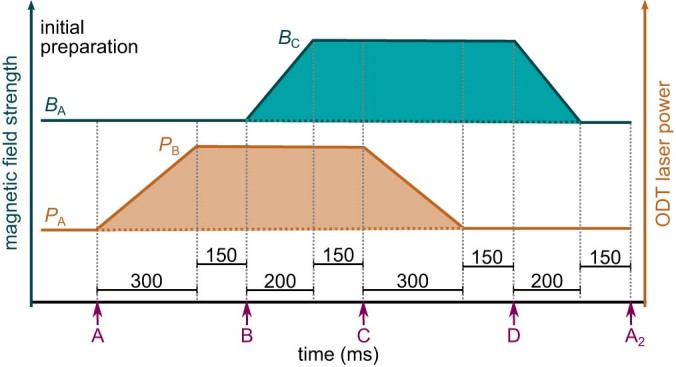
Sequence of the Pauli engine cycle. Magnetic field strength (cyan) and power of the ODT laser (orange). The imaging points during the cycle are marked with purple arrows (*A* denotes the first point of the cycle and *A*_2_ the last one after a full cycle).

The quantum Pauli cycle is implemented by alternating changes of the trap frequency through the dipole trap laser and of the interaction strength through the magnetic field close to the Feshbach resonance. Extended Data Fig. 1 illustrates the experimental sequence of a single cycle. After initial preparation of the molecular BEC, we compress the trap adiabatically, increasing the laser power of the ODT during 300 ms from *P*_A_ to *P*_B_. The trap frequencies in both radial directions increase with the square root of the laser power of the ODT whereas the trap frequency along the axial direction remains irrelevant compared with the trapping frequency of the magnetic field for all the powers used in this work. The resulting geometric mean trap frequencies are given in Extended Data Table 1. After a waiting time of 150 ms with constant trap frequency, we reach cycle point B. Then, we adiabatically increase the magnetic field strength linearly from *B*_A_ to *B*_C_ to change the interaction strength during the stroke B → C. Changing the magnetic field strength does not substantially alter the trap frequency in the axial direction. After a waiting time of 150 ms with constant magnetic field, we reach point C. In the next step, we ramp the ODT laser power to the initial value *P*_A_, expanding the gas (C → D) in the trap. Finally, we close the cycle with a second magnetic field ramp back to *B*_A_. After running an entire cycle, we verify that the measurement point A_2_ is equivalent to point A. In all of the measurement series, we have *B*_A_ < *B*_C_, *P*_A_ < *P*_B_ and, therefore, ${\bar{\omega }}_{{\rm{A}}} < {\bar{\omega }}_{{\rm{B}}}$. Extended Data Table 1 summarizes the experimental parameters for the three types of cycles that we run.

### Atom-number measurement

**Extended Data Fig. 2:**
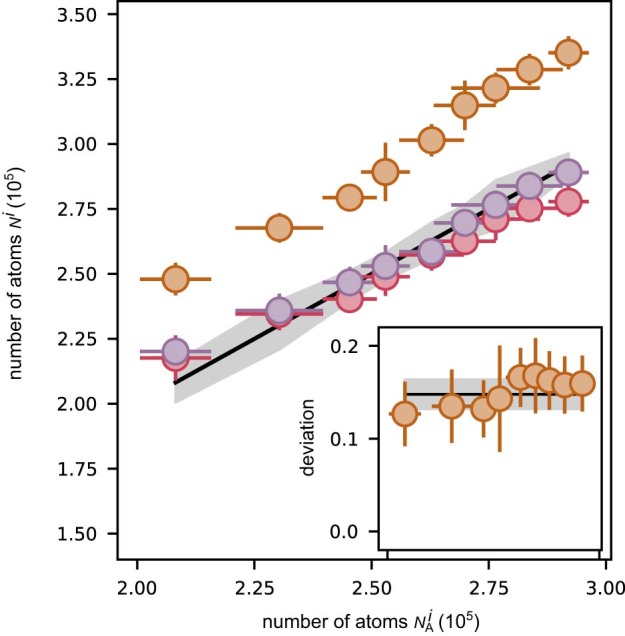
Determination of the correction factor of the number of atoms on resonance for the Pauli engine. Measured number of atoms of one spin state *N*^*i*^ in point B (red), C (orange), and return to point B_return_ after reaching point C (purple) in dependence of the number of atoms of one spin state ${N}_{{\rm{A}}}^{i}$ in point A. The measured number of atoms in point C cannot differ from the number of atoms in points B and B_return_, when the later are equal. Therefore, the measured number of atoms on resonance is higher than the actual number of atoms. Data points are averages of ten repetitions, uncertainties indicate one standard deviation. The inset shows the calculated deviation ${N}_{{\rm{C}}}/{N}_{{{\rm{B}}}_{{\rm{return}}}}-1$ (orange points) for the number of atoms on resonance in dependence of ${N}_{{\rm{A}}}^{i}$. The mean value of these deviations is 15 % and is visible as a black line, their standard deviation is the grey area. The *x*-axis of the inset spans the same range as the main axis.

To determine the number of atoms from in situ absorption pictures, we image the energetically higher-lying spin state *m*_*I*_ = 1 by standard absorption imaging on a charge-coupled device camera. This procedure is identical for all the regimes considered. Since the number of atoms of the spin state *m*_*I*_ = 0 is the same as for *m*_*I*_ = 1, the total number of atoms *N* (sum of atoms with spin up and down) is twice the number of atoms of the measured spin state *N*^*i*^. Importantly, in the case of a molecular BEC, the number of molecules is half the number of total atoms. We extract the column density from the measured absorption pictures. Adding up the density pixelwise and including the camera’s pixel area and the imaging system’s magnification, we obtain the number of atoms. We include additional laser-intensity-dependent corrections^51^. The imaging system has a resolution of 2.2 μm. The optical absorption cross-section changes its value throughout the BEC–BCS crossover, because it depends on the optical transition frequency^26^ which is, in turn, a function of the magnetic field strength. We determine the absorption cross-section for a magnetic field of 763.6 G and we use this value for further magnetic field strengths. This leads to the fact that the measured number of atoms on resonance is higher than the actual number of atoms. Therefore, we determine a correction factor to compensate this imperfection in independent measurements at different magnetic field strengths (Extended Data Fig. 2). We prepare an atomic cloud of varying atom number at *B*_A_ = 763.6 G. We then measure the atom number for this field at points A and B. We repeat the measurement for *B*_C_ and, to exclude atom loss, we ramp the field from *B*_B_ = *B*_A_ adiabatically to *B*_C_ and back to *B*_B_ before measuring. The atom numbers for *B*_B_ with and without the additional ramp to C are almost identical, from which we conclude that the number measured at *B*_C_ has to be the same. The correction factor is then calculated from the difference in atom number between these two data sets.

**Extended Data Fig. 3:**
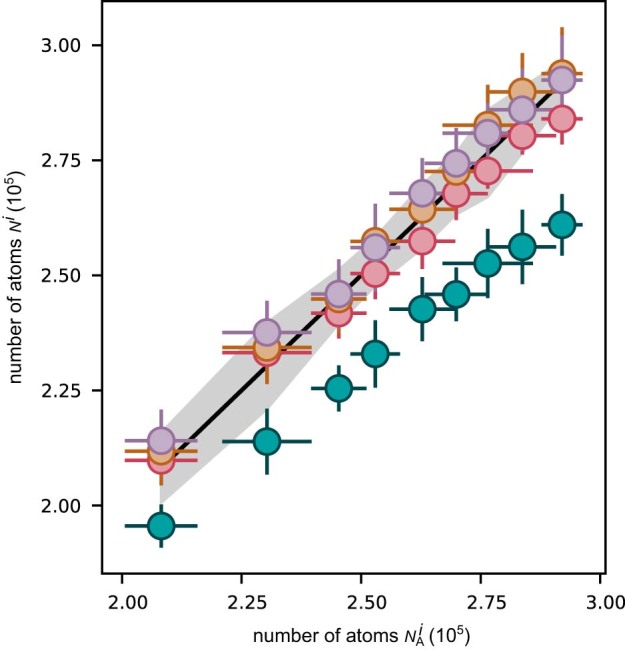
Atom-number correction in the Pauli cycle. Corrected number of atoms of one spin state *N*^*i*^ in the points of the cycle A (first point of the cycle, black solid line), B (red), C (orange), D (purple), and A_2_ (last point of the cycle, cyan) in dependence of the corrected number of atoms of one spin state ${N}_{{\rm{A}}}^{i}$ in point A. Experimental data belong to the Pauli engine in Fig. 4(a) and (c) with a compression ratio of ${\bar{\omega }}_{{\rm{B}}}/{\bar{\omega }}_{{\rm{A}}}=1.5$, the number of atoms is experimentally varied. Uncertainty is the standard deviation of 20 repetitions.

Extended Data Fig. 3 shows the measured number of atoms for one spin state *N*^*i*^ for the different points of the cycle of the Pauli engine as a function of the number of atoms of one spin state ${N}_{{\rm{A}}}^{i}$ in point A. The correction factors for different magnetic fields are included. In this way, we independently verify that we experimentally have a constant number of atoms during the cycle from A → D through B and C. Only the last stroke D → A_2_ suffers from atom losses of about 10%. These losses primarily occur when the quantum statistics are changed from Fermi–Dirac to Bose–Einstein in a shallow trap. The thermodynamics of adiabatic transfers through the crossover has been theoretically studied in ref. ^39^. During the change of statistics, bosonic molecules are formed. We attribute the losses to an excess-energy transfer between molecules during the ramping. Owing to the relatively low trap depth (needed for a high compression ratio), even small kinetic energies are sufficient to remove molecules from the trap.

The measured atom number has an uncertainty due to several mechanisms. First, the produced quantum gases feature a fluctuating number of particles due to uncontrolled technical fluctuations in, for example, cooling- and trapping-laser intensity or magnetic-field currents. But it is also due to physical statistical fluctuations, such as Poissonian fluctuations of the particle number during laser cooling or fluctuations occurring during the phase transition to a quantum fluid. These mechanisms cause fluctuations of the particle number of the produced ultracold clouds. Second, even for identical atom numbers, an additional uncertainty originates from the measurement process. We deploy resonant high-intensity absorption imaging^52,53^ on the transition ${| }^{2}{{\rm{S}}}_{1/2},{m}_{J}=-\,1/2,{m}_{I}=1\rangle \leftrightarrow {| }^{2}{{\rm{P}}}_{3/2},{m}_{J}=-\,3/2,{m}_{I}=1\rangle$ to acquire the column-integrated density distribution. Determining the accurate atom number from the resulting images requires precise control over the imaging pulse length and power, as well as calibration of the camera counts and effective absorption cross-section. In particular, fluctuations of the laser power after transmission through the imaging system in addition to camera noise are the main origin of statistical density uncertainties and dominate the uncertainty indicated in the measured data points. A systematic uncertainty is due to limited knowledge of the absorption cross-section. It can, in principle, be determined by the transition wavelength, but its value can change owing to optical pumping, the presence of magnetic fields or polarization effects, and was, therefore, determined by comparing theoretical density distributions of a BEC with measured ones^51^. The correction factor for our specific system was evaluated from a *χ*^2^ fit and has a systematic uncertainty of less than 10%. The resulting statistical atom-number fluctuations dominate the uncertainty for identical experimental parameters. We quantify them by the 1*σ* standard deviation of typically 20 identical realizations and indicate them as bars in equations (2) and (3) as well as in Figs. 2 and 4 around the mean atom number of the respective measurement series.

### Cloud-radii determination

**Extended Data Fig. 4:**
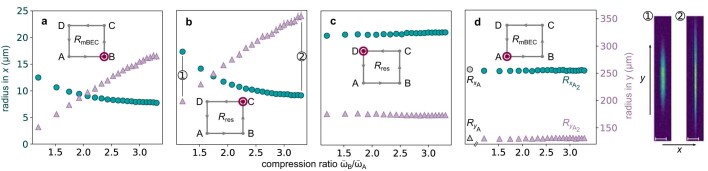
Realization of the Pauli engine. (a)-(d) Measured radii *R*_mBEC_ and ${R}_{{\rm{res}}}$ at each point of the cycle as a function of the compression ratio ${\bar{\omega }}_{{\rm{B}}}/\,{\bar{\omega }}_{{\rm{A}}}$ for constant number of atoms ${N}_{{\rm{A}}}^{i}=2.5\times 1{0}^{5}$. Radii are extracted by fits of the density profiles with equations (2) or (3). Radii are shown in *x*-direction (cyan circles) and *y*-direction (purple triangles). Experimental data are the mean value of 20 repetitions and the corresponding error bars indicate the propagation of the uncertainty of standard deviation of the measurement parameters. The insets show schematics of the cycle, where the point at which the measurement is taken is highlighted (purple). In (d), we compare the experimental values of the radii for A (first point of the cycle) in grey with the last point of the cycle A_2_ in the *x* (*y*) direction. The trap frequency ${\bar{\omega }}_{{\rm{B}}}$ has no influence on the first measurement point A and ${\bar{\omega }}_{{\rm{A}}}$ is constant for the different compression ratios. Therefore, this setting is only measured once with 20 repetitions. The experimental radii in A_2_ (last point of the cycle) are measured after running a full cycle (data points). On the right two exemplary absorption pictures at point C are shown.

We obtain line densities of the quantum gases by integrating the column-integrated density distributions of the two-dimensional (2D) absorption pictures *n*_2D_(*x*, *y*) = ∫ *n*_3D_(*x*, *y*, *z*) d*z* along the *x* and *y* directions separately. We fit these distributions with a 1D-integrated Thomas–Fermi profile. The Thomas–Fermi profiles are different for the interaction regimes throughout the BEC–BCS crossover. In the Thomas–Fermi limit, the in situ density distribution of a molecular BEC has the shape^9^

2 $${n}_{{\rm{mBEC}}}\propto {\left(1-\frac{{x}^{2}}{{R}_{{{\rm{mBEC}}}_{x}}^{2}}\right)}^{2},$$

where ${R}_{{{\rm{mBEC}}}_{x}}$ is the Thomas–Fermi radius of the atomic cloud in the *x* direction. For a resonantly interacting Fermi gas right at ${B}_{{\rm{res}}}$, the density profile ${n}_{{\rm{res}}}$ in the *x* direction can be written as^34^

3 $${n}_{{\rm{res}}}\propto {\left(1-\frac{{x}^{2}}{{R}_{{{\rm{res}}}_{x}}^{2}}\right)}^{5/2},$$

with the rescaled Thomas–Fermi radius ${R}_{{{\rm{res}}}_{x}}$ in the *x* direction. Therefore, we determine the measured cloud radii in both regimes by fitting the measured density profiles with the appropriate line shapes. The extracted radii in the *x* and *y* directions are shown in Extended Data Fig. 4. Our experimental set-up does not allow measurements of the radii *R*_*z*_ in the *z* direction. For this radial direction, however, we use the measured radius *R*_*x*_ in the *x* direction (radial) and correct the value with the corresponding ratio of the trap frequencies *R*_*z*_ = (*ω*_*x*_/*ω*_*z*_)*R*_*x*_ (refs. ^46,54^).

### Temperature measurements

We determine the temperature of the molecular BEC by means of a bimodal fit of the density profile (for more details, see ref. ^32^) at a magnetic field strength of 680 G. This value of *B* is chosen by considering the following trade-off: at lower magnetic fields, losses in the atom clouds are too high for quantitative temperature determination, whereas for higher magnetic fields, the condensate and thermal parts are not well separated, which complicates a bimodal fit.

**Extended Data Fig. 5:**
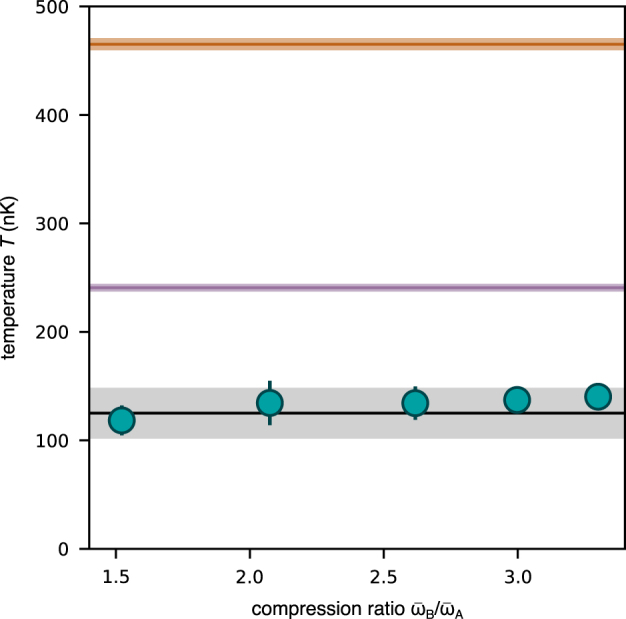
Measured temperature *T* for point A after ramping the magnetic field directly to 680 G (black line). Uncertainty is the standard deviation of ten repetitions (grey area). The cloud is compressed by increasing the mean trap frequency in stroke A → B. Afterwards, inverting the cycle direction leads again to point A. There, the temperature measurement is repeated after ramping the magnetic field to 680 G (cyan data points). Error bars show the standard deviation of ten repetitions. For comparison, critical temperature (purple solid line) and Fermi temperature (orange solid line) are depicted for point A. Shaded area shows the uncertainty and is of the order of the line thickness.

Owing to three-body recombination losses in the range between 550 G and 750 G (ref. ^55^), molecule losses in the cloud are already significant at 680 G. To avoid these losses, we choose a field of 763.6 G as the start of the Pauli cycle. To determine the temperature in an independent measurement, we ramp the field to 680 G during 200 ms and determine the temperature there. However, since decreasing the magnetic field value adiabatically throughout the BEC–BCS crossover increases the temperature *T* of the gas^39,48^, the measured temperature at 680 G is an upper bound for the temperature at point A and beyond. For the work stroke between points A and B, we increase the mean trap frequency. We observe that the reduced temperature *T*/*T*_F_ of the gas does not show significant changes during these work strokes. The temperature *T* does not change after ramping the trapping frequency back and forth. Because of this, we determine the temperature of the cloud, as mentioned above, with a bimodal fit for the two settings. First, we ramp the magnetic field from 763.6 G (value at point A) to 680 G to determine the temperature there. Second, we restart the sequence and run the cycle until point B. Afterwards, we change the direction of the cycle and go directly back to point A. A magnetic field sweep to 680 G allows again a temperature measurement. Extended Data Fig. 5 shows that the temperatures of the cloud after reversal to point A (cyan points) lie within the error (grey shaded area) of the initial temperature at point A (black line). We observe that an increase in temperature of less than 10% takes place for higher ratios of the mean trapping frequency, which can be neglected in the analysis (see below).

The method described above holds for atomic clouds below the critical temperature. For a thermal gas, we can directly determine the temperature of the magnetic field at the cycle point. At finite temperatures, density profiles of thermal clouds can be approximated with a classical Boltzmann distribution. The temperature *T*_*j*_ can be calculated dependent on direction *j* of the cloud ${T}_{j}=(m{\bar{\omega }}^{2}{{\sigma }}_{j}^{2})/{k}_{{\rm{B}}}$ (ref. ^46^), with *σ*_*j*_ as width of a Gaussian determined by fitting the 1D density profiles with ${n}_{{\rm{thermal}}}\propto \exp (-{j}^{2}/2{{\sigma }}_{j}^{2})$. In the case of molecular bosons, we use 2*m* for the mass instead of *m*. Hence, interactions directly influence the width of the cloud; we interpret this temperature for our experiment as an approximated temperature.

### Energy calculation

The calculation of the total energy *E*_mBEC_ of a molecular BEC is based on the Gross–Pitaevskii equation in the Thomas–Fermi limit for a harmonic trapping potential and for zero temperature. This energy consists of two parts, the kinetic energy *E*_kin_ and the energy in the Thomas–Fermi limit *E*_TF_, which takes into account the oscillator and interaction energies^46,54^

4 $$\begin{array}{l}{E}_{{\rm{mBEC}}}\,=\,{E}_{{\rm{TF}}}+{E}_{{\rm{kin}}}\\ \,\,=\,\left(\frac{{\mu }_{{\rm{mBEC}}}}{7}+\frac{{\hbar }^{2}}{4m{R}_{{\rm{mBEC}}}^{2}}\,\left[{\rm{ln}}\frac{{R}_{{\rm{mBEC}}}}{1.3{a}_{{\rm{ho}}}}+\frac{1}{4}\right]\right)\frac{5N}{2},\end{array}$$

where *R*_mBEC_ is the geometric mean radius of the cloud. The chemical potential is given by

5 $${\mu }_{{\rm{mBEC}}}=\frac{\hbar \bar{\omega }}{2}{\left(\frac{15\frac{N}{2}{a}_{{\rm{dd}}}}{{a}_{{\rm{ho}}}}\right)}^{2/5},$$

where *a*_dd_ = 0.6*a* is the *s*-wave scattering length for molecules^8^ and ${a}_{{\rm{ho}}}=\sqrt{\hbar /(2m\bar{\omega })}$ is the oscillator length. The radii and molecule numbers are extracted from the absorption pictures for the considered interaction strengths. When including the molecular energy, which gives the amount of energy needed to dissociate a molecule into two atoms, the total energy of the molecular BEC *E*_mBEC,m_ is given by

6 $${E}_{{\rm{mBEC}},{\rm{m}}}={E}_{{\rm{mBEC}}}-\frac{N}{2}\frac{{\hbar }^{2}}{m{a}^{2}}.$$

On resonance, the scattering length diverges and the binding energy vanishes. When we tune the magnetic field to resonance, the system evolves into a strongly interacting unitary Fermi gas. Its total energy ${E}_{{\rm{res}}}$ is related to that of an ideal binary Fermi gas through scaling with the universal constant $\sqrt{1+\beta }$

7 $${E}_{{\rm{res}}}=\frac{3}{4}\sqrt{1+\beta }{E}_{{\rm{F}}}N,$$

where ${E}_{{\rm{F}}}={(3N)}^{1/3}\hbar \bar{\omega }$ is the Fermi energy^10,33,34,56^. This zero-temperature expression provides a good approximation for *T*/*T*_F_ < 0.2 as supported by the energy measurements on resonance reported in refs. ^56–58^ (the low-*T* measurements are almost independent of *T* in the mentioned range). It should be noted that owing to the different temperature dependency of the entropy of the bosonic (*S*^bosons^ ∝ *T*^3^) and fermionic (*S*^fermions^ ∝ *T*) trapped gases, a decrease in the temperature is expected when performing an adiabatic sweep from the molecular BEC side to the BCS side and to unitarity. This internal temperature decrease is another indication that the operation of the Pauli engine is not driven by thermal energy. Since the condition *T*/*T*_F_ < 0.2 is fully satisfied for points C and D in all of our experiments the zero-*T* formulas on resonance are highly accurate.

**Extended Data Fig. 6:**
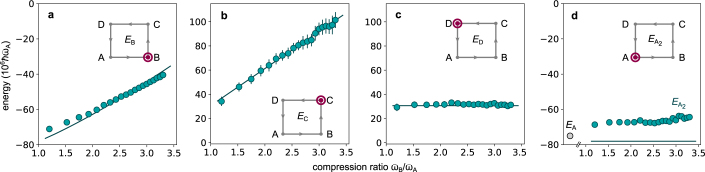
Realization of the Pauli engine. Energies (a) *E*_B_, (b) *E*_C_, (c) *E*_D_, and (d) *E*_A_ (first point of the cycle) and ${E}_{{{\rm{A}}}_{{\rm{2}}}}$ (last point of the cycle) at each point of the cycle as a function of the compression ratio ${\bar{\omega }}_{{\rm{B}}}/\,{\bar{\omega }}_{{\rm{A}}}$ for constant number of atoms *N*^*i*^ ≈ 2.5 × 10^5^. Experimental data (cyan points) are the mean value of 20 repetitions and the corresponding error bars indicate the propagation of uncertainty of standard deviation of the measurement parameters. The experimental data fits well to numerical calculations (cyan solid line), except for point A_2_ in (d) because of atom losses. The insets show schematics of the engine with the individually points of the engine highlighted in purple. In (d) the experimental value of the energy *E*_A_ is shown in grey for comparison to A_2_. The trap frequency ${\bar{\omega }}_{{\rm{B}}}$ has no influence in the first measurement at point A, and ${\bar{\omega }}_{{\rm{A}}}$ is constant for the different compression ratios. Therefore, *E*_A_ is only measured once with 20 repetitions.

The obtained experimental values were contrasted with those obtained using well-known formulas for BECs^46,54^ in the Thomas–Fermi limit for interacting bosons adapted as previously explained for composite bosons made up of fermions of opposite spin states^5,6,59^. In these cases, we use the theoretically expected radius and a constant number of particles during the cycle equal to the experimental number of particles at point A. Using equations (4), (5) and ${R}_{{\rm{mBEC}}}={(9{\hbar }^{2}Na/(8{m}^{2}{\bar{\omega }}^{2}))}^{1/5}$ in equation (6), we obtain the following energy for the molecular BEC regime at zero temperature.

8 $${E}_{{\rm{mBEC}},{\rm{m}}}\,=\,\frac{N}{2}\left\{\frac{5}{14}\hbar \bar{\omega }{\left(\frac{9\frac{N}{2}a}{\sqrt{\frac{\hbar }{2m\bar{\omega }}}}\right)}^{\frac{2}{5}}+\frac{5}{4}\frac{{\hbar }^{2}}{m{\left({\left({\left(\frac{{\hbar }^{2}}{2m\bar{\omega }}\right)}^{2}9\frac{N}{2}a\right)}^{\frac{1}{5}}\right)}^{2}}\left[{\rm{ln}}\left(\frac{10}{13}{\left(\frac{9\frac{N}{2}a}{\sqrt{\frac{\hbar }{2m\bar{\omega }}}}\right)}^{\frac{1}{5}}\right)+\frac{1}{4}\right]\right\}-\frac{N}{2}\frac{{\hbar }^{2}}{m{a}^{2}},$$

where the first two terms are related to the energy of bosonic particles in a trap including the interaction between bosons (the molecules interact with each other by means of a contact interaction^46^ that can be quantified using the interaction strength^8^ *g* = 1.2π*ħ*^2^*a*/*m*) and the last term is the contribution of the molecular energy of each of the pairs. For the zero-*T* calculations of the ground-state energy of the bosonic system, we also numerically solve the Gross–Pitaevskii equation with the bosonic interaction in terms of the dimer–dimer scattering length. Owing to the range of the experimental parameters, the Thomas–Fermi approximation holds in all our experiments in the molecular BEC regime; therefore, the numerical results are the same as those obtained when using equation (8) (refs. ^46,54^). Extended Data Fig. 6 shows the experimental and theoretical energies for each point of the Pauli cycle for the data set of Fig. 3 (point A denotes the first point of the cycle and point A_2_ the last point after a full cycle).

For low but non-zero temperature, the Thomas–Fermi approximation to the Gross–Pitaevskii equation reads^46^

9 $$\begin{array}{l}{E}_{{\rm{mBEC}},{\rm{m}}}^{T}\,=\,\frac{N}{2}\hbar \bar{\omega }{\left(\frac{\frac{N}{2}}{\zeta (3)}\right)}^{\frac{1}{3}}\left\{\frac{3\zeta (4)}{\zeta (3)}{\left(\frac{T}{\frac{\hbar \bar{\omega }}{{k}_{{\rm{B}}}}{\left(\frac{\frac{N}{2}}{\zeta (3)}\right)}^{\frac{1}{3}}}\right)}^{4}+\frac{\zeta {(3)}^{\frac{1}{3}}}{14}{\left(9{\left(\frac{N}{2}\right)}^{\frac{1}{6}}\frac{a}{\sqrt{\frac{\hbar }{2m\bar{\omega }}}}\right)}^{\frac{2}{5}}\right.\\ \,\,{\left(1-{\left(\frac{T}{\frac{\hbar \bar{\omega }}{{k}_{{\rm{B}}}}{\left(\frac{\frac{N}{2}}{\zeta (3)}\right)}^{\frac{1}{3}}}\right)}^{3}\right)}^{\frac{2}{5}}\left(5+16{\left(\frac{T}{\frac{\hbar \bar{\omega }}{{k}_{{\rm{B}}}}{\left(\frac{\frac{N}{2}}{\zeta (3)}\right)}^{\frac{1}{3}}}\right)}^{3}\right)\}-\frac{N}{2}\frac{{\hbar }^{2}}{m{a}^{2}}.\end{array}$$

According to Tan’s generalized virial theorem^10,42,60–62^, the energy for a cigar-shaped trapped Fermi gas is given by $$E=Nm(2{\omega }_{r}^{2}{\langle r}^{2}\rangle \,+$$$${\omega }_{z}^{2}{\langle z}^{2}\rangle )-{\hbar }^{2}{\mathcal{I}}/(8{\rm{\pi }}ma)$$, where *r* and *z* stand for the radial and axial coordinates, respectively, and $${\mathcal{I}}$$ is the contact (a measure of the probability for two fermions with opposite spins being close together^10^). This expression is valid for any value of the scattering length *a* and for any temperature *T*. On resonance, that is, at unitarity, we have 1/*k*_F_*a* = 0 and a finite $${\mathcal{I}}$$. Therefore the virial theorem reduces to the case presented by Thomas et al. in refs. ^40,41^, that is, $$E=Nm(2{\omega }_{r}^{2}{\langle r}^{2}\rangle +{\omega }_{z}^{2}{\langle z}^{2}\rangle )$$. In the weak coupling limit for the molecular BEC side, the contact reduces to $${\mathcal{I}}\approx 4\pi N/a$$, in which case the last term of the virial expression gives the total molecular energy and the virial energy reduces to equations (8) and (9) (refs. ^42,46^). All of this means that the expression −*ħ*^2^/(*ma*^2^) for the molecular term holds even for *T*/*T*_F_ ≈ 0.7 as long as the experiments are realized in the deep molecular BEC regime. For our magnetic fields, this condition is fulfilled for the Pauli engine and Feshbach cycle  as well as for the thermal cycle. Furthermore, measurements of the molecular energy were presented in ref. ^4^ for *T*/*T*_F_ ≈ 0.15, which show that the formula −*ħ*^2^/(*ma*^2^) holds for *T*/*T*_F_ ≈ 0.2 in a complete *B* sweep from molecular BEC to unitarity.

In the high-*T* regime the interactions are negligible and the distributions can be approximated by Maxwell–Boltzmann distributions. When the thermal energy *k*_B_*T* is large enough to break all the pairs, the energy can be approximated by that of an ideal classical gas of atomic particles for both magnetic fields. In this case, the temperature in the work strokes does not change and there is no work output. When the thermal energy is below the binding energy, the energy for a magnetic field below resonance corresponds to a classical gas of molecules with mass 2*m* whereas the energy at the resonant field is given by a classical gas of atoms in a harmonic trap. The molecular term cancels in the total work output giving $W=3{k}_{{\rm{B}}}N\left({T}_{{\rm{A}}}/2-{T}_{{\rm{D}}}+{T}_{{\rm{C}}}-{T}_{{\rm{B}}}/2\right)$. The temperature of the gas is obtained through a Gaussian fitting of the density profile leading to ${k}_{{\rm{B}}}T=m{\bar{\omega }}^{2}{\sigma }^{2}$, where *σ* is the width of the fitted density profile. Since ${\bar{\omega }}_{{\rm{D}}}={\bar{\omega }}_{{\rm{A}}}$ and ${\bar{\omega }}_{{\rm{C}}}={\bar{\omega }}_{{\rm{B}}}$ and the mass of the molecules is twice that of one of the atoms, we find ${T}_{{\rm{A}}}/2={({\sigma }_{{\rm{A}}}/{\sigma }_{{\rm{D}}})}^{2}{T}_{{\rm{D}}}$ and ${T}_{{\rm{B}}}/2={({\sigma }_{{\rm{B}}}/{\sigma }_{{\rm{C}}})}^{2}{T}_{{\rm{C}}}$, that is, $W=3{k}_{{\rm{B}}}N\left\{\left({\sigma }_{{\rm{A}}}^{2}\,/{\sigma }_{{\rm{D}}}^{2}-1\right){T}_{{\rm{D}}}+\left(1-{\sigma }_{{\rm{B}}}^{2}\,/{\sigma }_{{\rm{C}}}^{2}\,)\right){T}_{{\rm{C}}}\right\}$. The work vanishes for *σ*_A_ = *σ*_D_ and *σ*_B_ = *σ*_C_. In our experiments, the widths do not show significant statistical differences and therefore the work output vanishes for the engine running in the high-*T* regime.

Let us now detail each one of the theoretical curves presented in the main text. Based on Tan’s generalized virial theorem, we calculate the theoretical trap energy of Fig. 2 by means of equation (9) without the molecular term and using the experimental temperatures at points A and B, whereas for the points C and D we use the energy for zero *T* at unitarity given by equation (7). The vanishing efficiency for the thermal case is expected from the classical gas argument of the previous paragraph. The cyan curves in Fig. 3 arise from numerical calculations of the Gross–Pitaevskii equation. The black dashed curves in Fig. 3e,f were also calculated by solving numerically the Gross–Pitaevskii equation and yield the same results as an ideal molecular gas of point-like bosons having an infinite binding energy, which leads to ${W}_{{\rm{non}}-{\rm{interacting}}}={(3N)}^{4/3}\sqrt{1+\beta }\hbar ({\bar{\omega }}_{{\rm{B}}}-{\bar{\omega }}_{{\rm{A}}})/4$ and *η*_non-interacting_ = 0. The purple curves in Fig. 3e,f correspond to the results obtained for ideal Bose and Fermi gases in the highly degenerate regime and lead to

10 $${W}_{{\rm{ideal}}}={(3N)}^{4/3}\hbar ({\bar{\omega }}_{{\rm{B}}}-{\bar{\omega }}_{{\rm{A}}})/4$$

and

11 $${\eta }_{{\rm{ideal}}}=1-{\bar{\omega }}_{{\rm{B}}}/{\bar{\omega }}_{{\rm{A}}}.$$

The ideal efficiency is similar to the maximum Otto efficiency^25^. Compared with these upper limits, we find that the experimental system shows reduced values but of the same order of magnitude. Our model allows us to predict the performance for different magnetic field values in the molecular BEC regime. The grey curves in Fig. 3e,f and the theoretical curves of Fig. 4 follow from equations (8) and (7). Since the latter calculations rely on zero*-T* formulas, we interpret their results as an upper bound or limit for the work output and efficiency. To show that this estimation is accurate, we need to consider two aspects. First, for the two on-resonance points (C, D), we consider the zero-*T* calculations to be a good approximation because of the aforementioned decay in the temperature between the molecular BEC regime and unitarity in the adiabatic sweep. Owing to the dominance of the molecular term on the molecular BEC side, the efficiency obtained when including the temperature overlaps with that obtained within the zero-*T* approximation. For the data of Fig. 4, the zero-*T* calculations give an efficiency of about 0.07(2) while the finite-*T* formulas give an efficiency of about 0.05(3). A null hypothesis test for the mean difference gives a *P* value of 0.1. Setting the usual significance of *α* = 0.05, the null hypothesis of equal efficiencies cannot be rejected. Therefore, we conclude that the finite-*T* corrections have no notable effects on the efficiency of our engine.

Figure 3e,f shows that the work output is reduced with increased initial effective repulsion of the molecular BEC, whereas the efficiency increases. This scaling of the work output is a consequence of the competition between interactions among molecules in the initial molecular BEC state and the effect of changing the quantum statistics. For stronger initial effective repulsion, the molecular BEC cloud already exhibits a relatively large energy in the trap, so that the change of quantum statistics can only contribute a comparatively smaller amount of energy during the Pauli stroke. This suggests an optimal work output for an initially non-interacting molecular BEC. It is important to mention that even though the binding energy has no consequences for the work output (the *s*-wave scattering length does not change during the work strokes) it still has to be provided to the system during the Pauli stroke when dissociating a molecule into two atoms; hence, it must be included in the energy-cost calculation of the engine’s efficiency. This binding energy quickly grows as the magnetic field deviates from the resonance value and the associated energy cost quickly reduces the efficiency. For the experimentally inaccessible case of a non-interacting molecular BEC, this binding energy is so large that the efficiency of the Pauli cycle is essentially zero. These considerations point toward an optimal point of operation which might, additionally, be temperature dependent.

An important result that demonstrates the non-classical drive of the Pauli engine is reproduced by both experimental findings and the theoretical model: the work output as a function of the number of particles *W* ∝ *N*^*α*^ scales with a fitted exponent *α* = 1.73(18), which is close to the prediction of 1.4 given by theory (Fig. 4a). The exponent of the work output with particle number is different from the 1D case mentioned in the introduction due to a modified density of states, that is, the number of available single-particle states in the potential. Although a similar exponent is expected for the Feshbach-driven stroke which remains always in the molecular BEC regime, the efficiency for this engine is close to zero. Most importantly, the exponent is larger than one, which is the expected exponent for a non-interacting, classical gas^1,48,63^.

### Pressure and volume calculations

**Extended Data Fig. 7:**
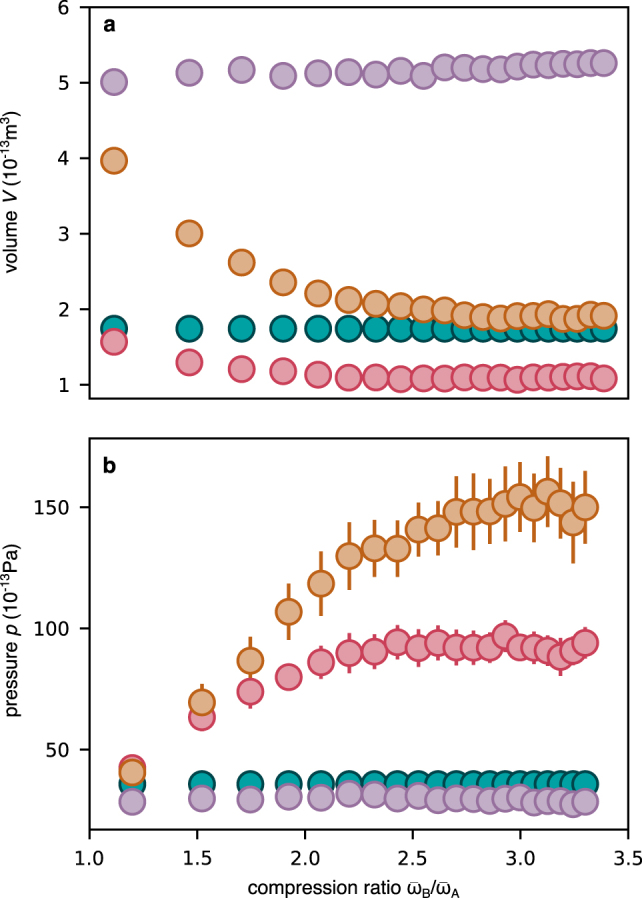
Pressure and volume for the Pauli engine. Volume *V* and pressure *p* (panel (a) and (b) respectively) for each point of the Pauli cycle: B (red), C (orange), D (purple), and A_2_ (last point of the cycle, cyan). The measurements corresponds to the same ones as in Fig. 3 of the main text. The data for the volume are the mean value of 20 repetitions with error bars that lie inside the point size, the error bars in the pressure indicate the propagation of uncertainty of standard deviation of the measurement parameters.

The pressure of the system is calculated as *p* = −∂*E*/∂*V*. As explained in the previous paragraph, the zero-*T* energy constitutes a good approximation for the system and, therefore, we calculate the pressure for *T* = 0. For computing the derivative at the experimentally obtained volumes, we use that, on the molecular BEC side, the theoretical volume is given by

12 $${V}_{{\rm{m}}{\rm{B}}{\rm{E}}{\rm{C}}}=\frac{4}{3}\pi {\left(\frac{9{\hbar }^{2}Na}{8{m}^{2}{\bar{\omega }}^{2}}\right)}^{\frac{3}{5}},$$

whereas, on resonance, the volume has a different dependence on the number of atoms and on the geometric mean of the trap frequency, and is given by

13 $${V}_{{\rm{r}}{\rm{e}}{\rm{s}}}=\frac{4}{3}\pi {(1+\beta )}^{\frac{3}{4}}\sqrt{24\frac{{\hbar }^{3}N}{{m}^{3}{\bar{\omega }}^{3}}},$$

(refs. ^34,46,54,64^). The experimental volume in both cases is computed as the volume of an ellipsoid having the radii obtained by means of equations (2) and (3). Extended Data Fig. 7 shows the corresponding values of volume *V* and pressure *p* as a function of the compression ratio. The *p–V* diagram in Fig. 2c is obtained using these experimental data (points) and by calculating the corresponding theoretical curves. For completeness, we also checked that the work output obtained from the area inside the *p–V* diagram −∫ *p* d*V* is in agreement with that shown in Fig. 3e.

A closer analogy with a quantum Otto cycle can be drawn using generalized parameters for *p* and *V* (refs. ^65,66^). It will be interesting in the future to extend this concept to ensembles with changing quantum statistics and to evaluate the performance of the Pauli engine according to this description.

## Online content

Any methods, additional references, Nature Portfolio reporting summaries, source data, extended data, supplementary information, acknowledgements, peer review information; details of author contributions and competing interests; and statements of data and code availability are available at 10.1038/s41586-023-06469-8.

## Figures and Tables

**Extended Data Table 1:**
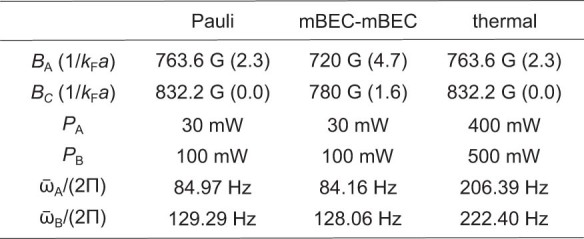
Experimental settings of the three types of cycles for which we vary the number of atoms

For the case in which we change the compression ratio ${\bar{\omega }}_{{\rm{B}}}$ of the Pauli engine, the power of the ODT laser *P*_B_ is iterated from 50 mW up to 1 W in steps of 50 mW. We extract the values of the *s*-wave scattering length *a* from ref. ^33^.

## Data Availability

All data of the figures in the manuscript and Methods are available in a Zenodo repository 10.5281/zenodo.7886857 (ref. ^67^).

## Code availability

The codes that support the findings of this paper are available from the corresponding author upon reasonable request.
